# Comparison of Different Buffers for Protein Extraction from Formalin-Fixed and Paraffin-Embedded Tissue Specimens

**DOI:** 10.1371/journal.pone.0142650

**Published:** 2015-11-18

**Authors:** Kaini Shen, Jian Sun, Xinxin Cao, Daobin Zhou, Jian Li

**Affiliations:** 1 Department of Hematology, Peking Union Medical College Hospital, Chinese Academy of Medical Sciences and Peking Union Medical College, Beijing, People's Republic of China; 2 Department of Pathology, Peking Union Medical College Hospital, Chinese Academy of Medical Sciences and Peking Union Medical College, Beijing, People's Republic of China; Moffitt Cancer Center, UNITED STATES

## Abstract

We determined the best extraction buffer for proteomic investigation using formalin-fixation and paraffin-embedded (FFPE) specimens. A Zwittergent 3–16 based buffer, sodium dodecyl sulfate (SDS)-containing buffer with/without polyethylene glycol 20000 (PEG20000), urea-containing buffer, and FFPE-FASP protein preparation kit were compared for protein extraction from different types of rat FFPE tissues, including the heart, brain, liver, lung, and kidney. All of the samples were divided into two groups of laser microdissected (LMD) and non-LMD specimens. For both kinds of specimens, Zwittergent was the most efficient buffer for identifying peptides and proteins, was broadly applicable to different tissues without impairing the enzymatic digestion, and was well compatible with mass spectrometry analysis. As a high molecular weight carrier substance, PEG20000 improved the identification of peptides and proteins; however, such an advantage is limited to tissues containing submicrograms to micrograms of protein. Considering its low lytic strength, urea-containing buffer would not be the first alternative for protein recovery. In conclusion, Zwittergent 3–16 is an effective buffer for extracting proteins from FFPE specimens for downstream proteomics analysis.

## Introduction

Formalin-fixation and paraffin-embedding (FFPE) enables the preservation and stabilization of tissue morphology, as well as the long-time storage of samples [1]. Therefore, FFPE tissues represent a valuable resource for retrospective molecular investigations. The antigen retrieval method proposed in 1991 allows the restoration of antigen immunoreactivity [2], thus making immunohistochemistry (IHC) an important technique for collecting antibody-based protein information from formalin-fixed samples. However, immunological methods rely on the availability and quality of the antibodies, which can be time-consuming and costly. In contrast, the advent of modern proteomics technology has revolutionized the field of clinical pathology through the comprehensive study of the protein constituents of a biological sample.

Proteomic analysis of FFPE specimens using mass spectrometry (MS) provides a promising method for studying disease-related protein differences, especially in clinical oncology. However, protein degradation and cross-linking induced by formaldehyde fixation was long assumed to hinder the maximum implementation of such tissues in proteomic studies [3]. The first reports demonstrating the ability to conduct MS-based proteomic analysis from FFPE tissues were by Hood et al. [4], Crockett et al. [5], and Palmer-Toy et al. [6]. Palmer-Toy et al. analyzed FFPE human temporal bone samples with a 2% sodium dodecyl sulfate (SDS) heating method, and retrieved 125 proteins from the spiral ligament sample. Currently, improvements in extraction buffer, pH, temperature, and pressure, have made the protein yield from FFPE samples comparable to that from fresh frozen tissue samples [7].

Among all of the analytical factors on proteomic profiling, the combination of detergent, reductant, and denaturant plays the most important role, since extraction buffer can induce protein unfolding, thereby increasing the protein accessibility from formalin-fixed samples. In 2012, Craven et al. [8] extracted about 2000 kinds of proteins from 5 cm^2^ renal cell carcinoma FFPE samples by applying 4% SDS-containing buffer. In 2010, Ostasiewicz et al. [9] reported that lysis buffer containing Tris-Hydrochloric acid, dithiothreitol (DTT), and SDS reproducibly resulted in 155 μg protein/mg liver tissue. With the addition of polyethylene glycol 20000 (PEG20000) to the aforementioned buffer, Wisniewski [9] identified 10,000 proteins from laser microdissected (LMD) colonic adenoma tissue. Despite the effectiveness of SDS-containing buffer, its incompatibility with enzymatic digestion and MS makes SDS removal necessary for the subsequent recognition of proteins. To avoid such an inconvenient step, urea was proposed as a substitute chaotropic reagent [10]. However, its limited lytic strength leaves certain proteins insoluble, particularly membrane proteins, thereby affecting subsequent digestion and peptide identification. In the Mayo Clinic, Zwittergent buffer was utilized in protein recovery to obtain the protein composition of involved tissue for the further typing of systemic amyloidosis [11]. Moreover, commercially available extraction kits were also applied to different tissues according to the manufacturer’s instructions [12].

Currently, both section and microdissected samples are used for proteomic analysis. However, until now, no extraction buffer was established that was applicable to both kinds of specimens. Additionally, no study has determined whether the effectiveness and efficiency of an extraction method are tissue-specific. In the current study, we compared five extraction buffers for the protein analysis of different kinds of rat LMD and FFPE tissue slices, including the heart, brain, liver, lung, and kidney. Our aim was to confirm the optimal extraction buffer for accurate identification of the largest number of proteins from samples processed by both microdissection and conventional sectioning of different types of tissues.

## Materials and Methods

### Rat FFPE tissues

Ten-week-old, male Sprague-Dawley rats were used in the present study. The rats were anesthetized intra-abdominally with chloral hydrate (5 ml/kg), and perfused intracardially with 0.9% saline followed by 4% formaldehyde. The brains, hearts, lungs, livers, and kidneys were carefully removed and fixed overnight in 4% formaldehyde at 4°C. A section of the organs was then embedded in paraffin and kept at room temperature until subsequent use. This study was carried out in strict accordance with the recommendations in the Guide for the Care and Use of Laboratory Animals of the National Institutes of Health. The protocol was approved by the Committee on the Ethics of Animal Experiments of Chinese Academy of Medical Sciences (Permit Number: XHDW-2013-034). All surgery was performed under chloral hydrate anesthesia, and all efforts were made to minimize suffering.

### Tissue preparation for liquid chromatography tandem MS

In total, 6 μm sections were cut from each FFPE block, mounted on glass slices, and deparaffinized by incubating through three changes of xylene for 5 min each. Next, the sections were rehydrated through a series of graded ethanol for 1 min each (100%, 90%, 80%, 70%), and then incubated in distilled water for at least 30 min. Two to six tissue sections were scraped from the slices as one sample, and mixed with different extraction buffers including: (1) extraction buffer containing 0.2% Zwittergent 3–16, 10 mM Tris, and 1 mM EDTA; (2) UPX universal extraction buffer taken from the FFPE-FASP kit (Thermo, USA); (3) extraction buffer containing 100 mM Tris, 100 mM DTT, and 4% SDS, pH 8.0; (4) extraction buffer containing 0.5% PEG20000, 100 mM Tris, 100 mM DTT, and 4% SDS, pH 8.0; and (5) extraction buffer containing 8 M urea, 2 M thiourea, 65 mM DTT, 83 mM Tris, and 4% CHAPS. To obtain microdissected samples, tissue slices were put under the light microscope and microdissected using the Leica LMD6500 system. Each microdissection was the same area of 1,000,000 μm^2^. Then, the tissue samples were resuspended in the different aforementioned buffers. The homogenates were heated at 100°C for 20 min and 60°C for 2 h, followed by 40 min of sonication in a water bath. All of the samples were reduced in 20 mM DTT at 37°C for 60 min, and alkylated with 25 mM iodoacetamide in the dark for 45 min at room temperature. Then the extracts were centrifuged for 10 min at 21,000×*g* at 4°C, and the supernatants were treated to reduce the detergent content using Pierce Detergent Removal Spin Columns (Thermo, USA) according to the manufacturer’s instruction. Next, the protein mixtures were transferred to spin ultrafiltration units of the nominal molecular weight cut-off of 10 kDa, and washed with 25 mM ammonium bicarbonate (NH_4_HCO_3_) three more times. Subsequently protein quantification was determined by the Bradford method. For each tissue sample, equal volumes of protein extracts, obtained from each FFPE sample using different extraction buffers, were mixed with SDS-PAGE buffer, incubated at 95°C for 5 min, and resolved on SDS-PAGE gels before staining with Brilliant blue G-250 (Solarbio, USA). Using a microwave-assisted protein enzymatic digestion protocol, proteins were directly digested in spin ultrafiltration units, using trypsin at an enzyme-to-protein ratio of 1:50 [13]. For each sample, the released peptides were collected and desalted with an oasis HLB 1 cc Vac Cartridge (Waters, USA). For microdissected specimens, digested peptides were desalted with ZipTip Micro-C_18_ pipette tips (Millipore, USA), after which they were further resuspended in 1% FA prior to liquid chromatography-tandem mass spectrometry (LC-MS/MS) analysis.

### LC-MS/MS analysis

The digested samples were analyzed using a self-packed RP C18 capillary LC column (75 μm×100 mm, 3 μm). The eluted gradient was 5%-30% buffer B1 (0.1% formic acid, 99.9% ACN; flow rate, 0.3 μL/min) for 20 min (LMD sample) or 50 min (conventional sectioned/non LMD sample). Each sample was run twice. An Orbitrap-LTQ-HCD mode was used to acquire the raw data. The MS data were acquired using the following parameters: ten data-dependent HCD MS/MS scans per full scan; full scans were acquired in Orbitrap at a resolution of 30,000; 30% normalized collision energy in HCD included internal mass calibration (445.120025 ions as lock mass with a target lock mass abundance of 0%), charge state screening (excluding precursors with an unknown charge state or +1 charge state), and dynamic exclusion (exclusion size list 500, exclusion duration 60 s).

### Data processing

All of the MS/MS samples were analyzed using Mascot (Matrix Science, UK; version 2.3.02). Mascot was set up to search the Swiss-Prot_rat (www.uniprot.org) database assuming the digestion enzyme was trypsin. Mascot was searched with a parent and fragment ion tolerance of 10 ppm and 0.1 Da. Carbamidomethyl of cysteine was specified as a fixed modification, whereas the oxidation of methionine was specified as a variable modification. Scaffold (Proteome Software, Portland; version 4.3.2) was used to validate MS/MS-based peptide and protein identification. Peptide identification was accepted at a 1% false positive rate at the protein level, and with at least two unique peptides. Proteins that contained similar peptides and could not be differentiated based on MS/MS analysis alone were grouped to satisfy the principles of parsimony. Proteins sharing significant peptide characteristics were grouped into clusters.

### Intensity-based absolute quantification of proteins

Protein abundances were estimated using the intensity-based absolute quantification (iBAQ) algorithm [14]. Briefly, the protein intensities were summarized from all identified peptide intensities by Progenesis LC-MS (v2.6, Nonlinear Dynamics, UK). iBAQ was obtained by dividing the protein intensities by the number of theoretically observable peptides. Since we did not include internal standards, iBAQ in current study was a relative quantification for comparison of protein extraction efficiency among different buffers.

### Statistical analysis

**Fig 1:**
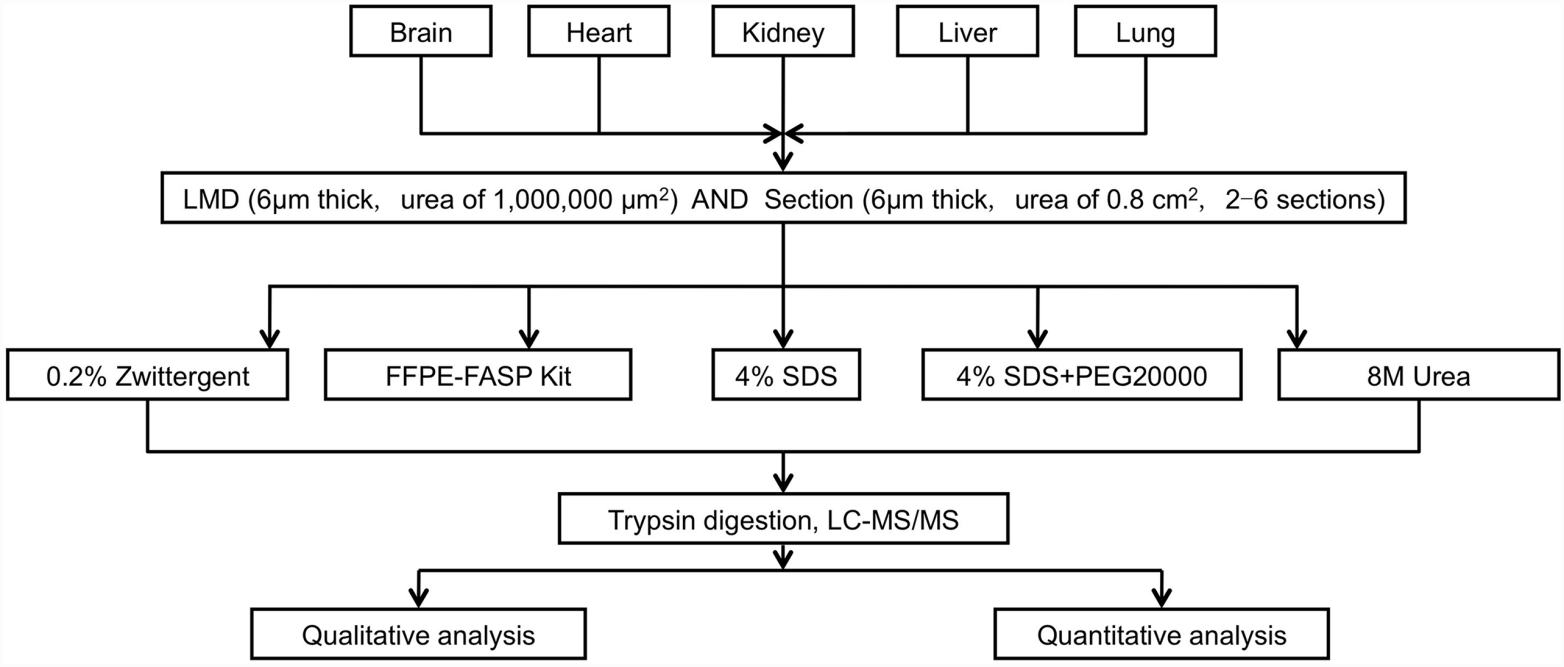
Workflow of the current study.

Differences in the spectrum, peptide, protein count, and protein iBAQ value among the five kinds of extraction buffers were tested using two-way analysis of variance (ANOVA). All of the tests were two-sided, and p values less than 0.05 were considered statistically significant. Statistical analyses were performed with SPSS version 20.0 software. Our workflow is displayed in Fig 1.

## Results

### LC-MS/MS results from LMD FFPE samples

**Fig 2:**
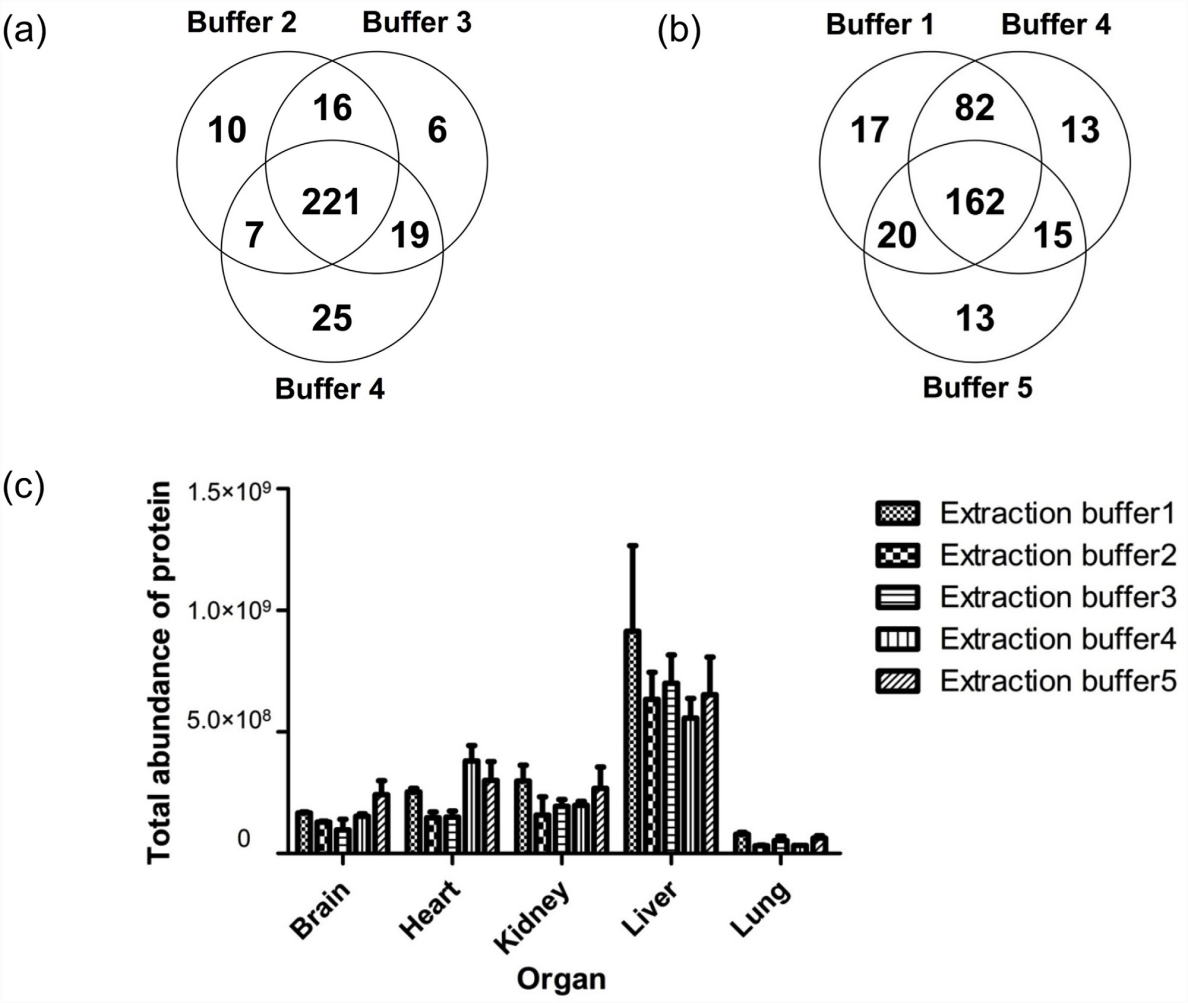
LMD/MS results from the five different extraction buffers. (a) Comparison of the three SDS-based extraction buffers (buffers 2, 3, 4). The number of unique identified proteins from the LMD kidney specimen was highest using buffer 4. (b) Comparison of the Zwittergent-containing buffer, the most efficient SDS-containing buffer (buffer 4), and the urea-containing buffer. The number of unique identified proteins from the LMD kidney specimen was highest using the Zwittergent-containing buffer. (c) Total iBAQ was comparable among buffers 1, 3, and 4, with the highest intensity found in proteins extracted using the Zwittergent-containing buffer.

Typical structural and functional proteins for each kind of rat organ, which were extracted using five kinds of buffers, were identified using Mascot search engines. In total, 44%-82% of proteins were identified with two or more peptides. The top five proteins based on spectra counts were summarized (S1 Table). The largest number of total spectra and identified peptides was from Zwittergent-containing and PEG20000-containing buffers, without a significant difference between each other (p = 0.454 and 0.844, respectively). The numbers of identified proteins were similar among buffers 1, 3, and 4 (Table 1), whereas a much lower number of peptides and proteins were extracted using the buffer from the FFPE-FASP Kit and the urea-containing buffer. For the lung and kidney samples, Zwittergent-containing buffer was a better choice, whereas PEG-containing buffer was more suitable for the brain and heart. SDS-based buffers (buffers 2, 3, 4) shared more than 80% proteins in common (Fig 2a). The numbers of unique identified proteins and peptides were both highest in the Zwittergent-containing buffer group. In Fig 2b, the LMD kidney specimen is shown as an example; the Zwittergent-based buffer led to the extraction of more unique proteins than the SDS-containing buffer. To further evaluate the digestion efficiency of different buffers, protein coverage were compared (S2 Table). Among different rat organs, average protein coverage of Zwittergent reached 9.98%-13.06%, with median coverage percent of 5.29%-9.10%, clearly superior than SDS-containing and urea-containing buffers. Since the total amount of proteins was relatively low, we used iBAQ as a quantitative parameter to assess the efficiency of the different extraction buffers. Total iBAQ was comparable among buffers 1, 3, and 4, with highest intensity in proteins extracted with the Zwittergent-containing buffer (Fig 2c). In addition, with regard to protein abundance, the Zwittergent-containing buffer was generally applicable to all five kinds of tissues.

**Table 1:** Comparison of proteins identified using five different extraction buffers after LMD/MS analysis.

	Buffer 1	Buffer 2	Buffer 3	Buffer 4	Buffer 5
Brain	128±19.8	105±15.6	81±25.5	141±1.4	82±14.1
Heart	104±12.7	80±28.3	83±5.7	112±4.9	67±5.7
Kidney	199±2.8	132±34.6	162±18.4	136±41.0	86±36.8
Liver	169±20.5	180±4.2	184±27.6	167±24.0	110±43.8
Lung	105±31.1	62±0.0	78±13.4	84±8.5	39±2.8
Mean	141	112	117	128	77
p*	-	0.046	0.150	0.679	<0.001

*: Extraction buffers 2–5 compared to extraction buffer 1.

### LC-MS/MS Results from FFPE Tissue Slices

**Fig 3:**
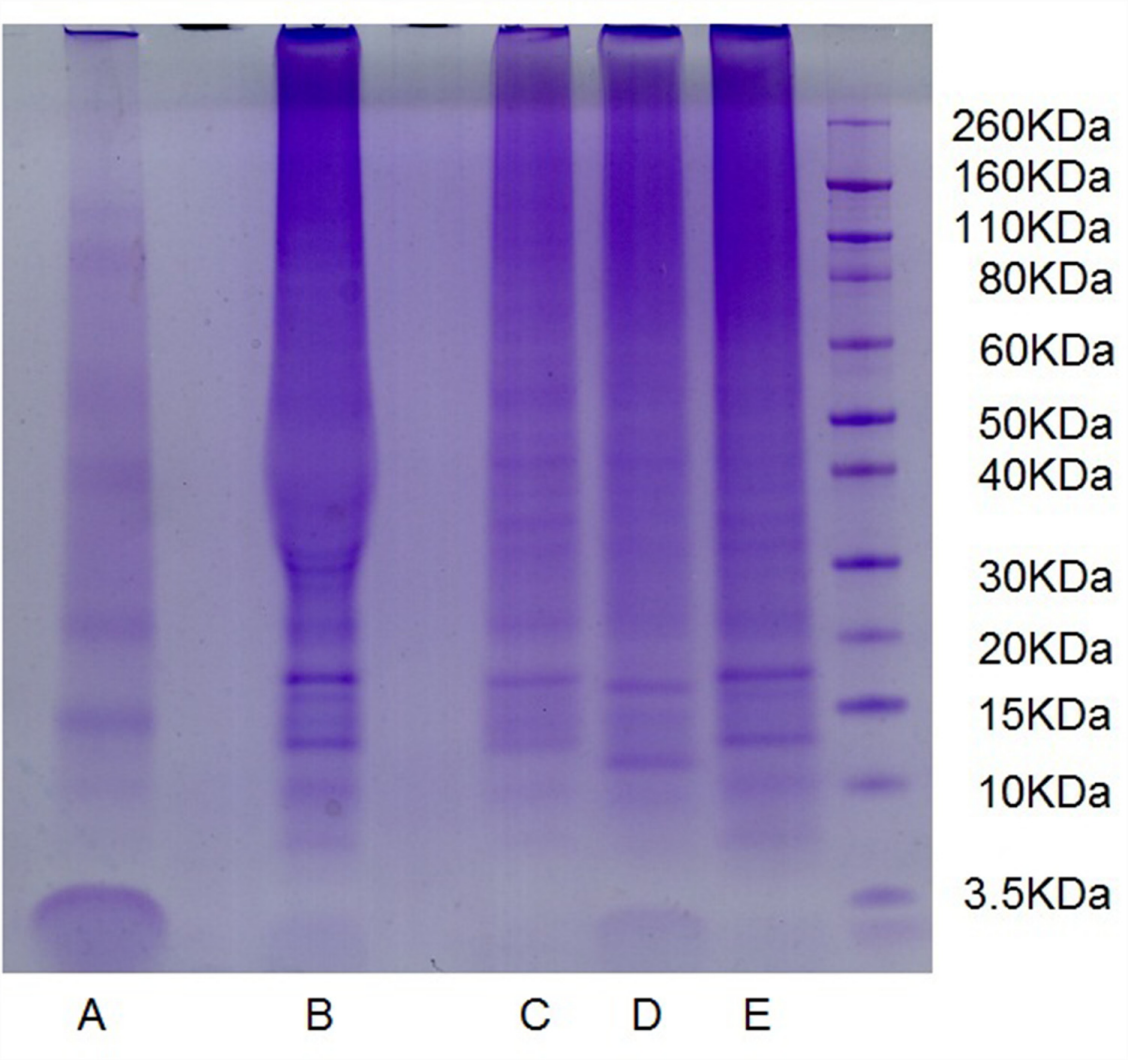
Comparison of the electrophoretic patterns of proteins extracted from the kidney FFPE sample using different buffers. a, extraction buffer 1; b, extraction buffer 4; c, extraction buffer 3; d, extraction buffer 5; e, extraction buffer 2.

Table 2 summarized the protein yield in extracts from FFPE tissue samples extracted using different kinds of buffers. The amount of protein extracted using the Zwittergent-containing buffer was about 1/5-1/2 less than that using the other buffers. In the protein extracts obtained using the buffer from the FASP Kit, the SDS-containing buffer without PEG20000, and the urea-containing buffer, more precise single lanes were visualized, whereas lanes from the extracts obtained using Zwittergent 3–16 were weaker and less distinct with smearing. However, there appeared to be some unique protein bands migrating at 60–160 kDa utilizing Zwittergent 3–16 buffer (Fig 3). On the whole, the pattern of proteins resolved on SDS-PAGE gels was of low quality, with a greater abundance of lower molecular weight proteins ranging from 10 to 50 kDa.

**Table 2:** Protein yield (μg) of extracts from FFPE tissue slices using different extraction buffers.

	Buffer 1	Buffer 2	Buffer 3	Buffer 4	Buffer 5
Brain	37.0	44.5	48.2	76.8	39.4
Heart	52.8	101.6	140.8	276.0	221.6
Kidney	53.8	254.3	215.8	317.0	225.4
Liver	35.0	38.6	36.1	66.9	37.5
Lung	32.8	58.4	137.6	243.2	164.8

**Fig 4:**
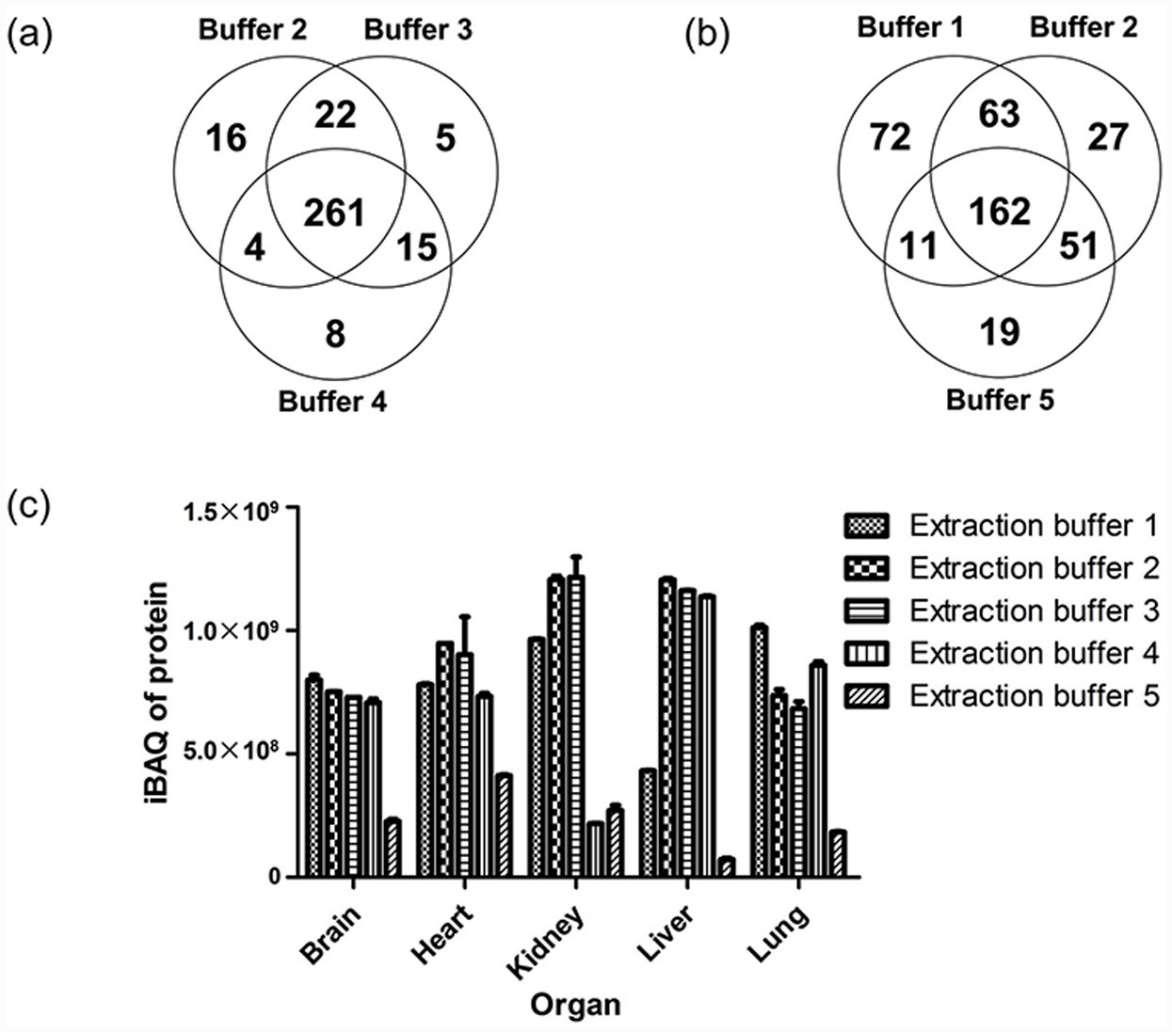
MS results from FFPE samples using five different extraction buffers. (a) When comparing the three SDS-containing extraction buffers (buffers 2, 3, 4), the number of unique identified proteins from the heart FFPE specimens was highest in the buffer 2 group. (b) When comparing the Zwittergent-containing buffer, the most efficient SDS-containing buffer (buffer 2), and the urea-containing buffer, the number of unique identified proteins from the heart FFPE specimen was highest in the Zwittergent-containing buffer group. (c) Total protein iBAQ of the Zwittergent-containing buffer was significantly lower than that of buffers 2 and 3, and was higher than that of buffers 4 and 5.

All of the FFPE tissue samples were analyzed in duplicate. Top five proteins according to spectra counts were displayed (S3 Table). Extraction buffers 1, 2, and 3 were clearly more proficient than the other buffers, when the number of spectra, peptides, and proteins (Table 3) were compared. The numbers of unique identified proteins and peptides were highest in the Zwittergent-containing buffer group, which is illustrated in Fig 4b using the FFPE heart tissue sample as an example. In the SDS-based buffers (buffers 2, 3, 4), 85%-90% of the identified proteins were the same (Fig 4a). For the brain, heart, and kidney, Zwittergent was the most efficient extraction buffer. Average protein coverage percent of Zwittergent ranged from 12.33% to 17.98%, while SDS-based buffer from 7.28% to 15.37%, urea-based buffer from 1.77% to 5.25% (S4 Table), proving the high digestion efficiency of Zwittergent regardless of tissue type.

**Table 3:** Total number of identified proteins from tissue specimens using five different extraction buffers after LC-MS/MS analysis.

	Buffer 1	Buffer 2	Buffer 3	Buffer 4	Buffer 5
Brain	426±45.3	405±3.5	388±3.5	344±7.8	239±5.7
Heart	234±3.5	215±4.2	204±13.4	201±0.7	135±3.5
Kidney	442±5.7	413±33.2	398±2.8	286±51.6	177±53.7
Liver	374±3.5	420±0.0	397±0.7	356±2.1	80±33.9
Lung	334±6.4	358±26.2	372±10.6	388±4.2	200±0.0
Mean	362	362	351	315	166
p*	-	1.000	0.809	<0.001	<0.001

*: Extraction buffers 2–5 compared to extraction buffer 1.

With regard to the protein iBAQ, Zwittergent 3–16 was less efficient than the buffer from the FASP Kit and the SDS-containing buffer without PEG20000 (p<0.001), but was more efficient than the SDS-containing buffer with PEG20000 and the urea-based buffer (p = 0.004 and p<0.001, respectively) (Fig 4c).

## Discussion

### Significance of the protein extraction buffer

Currently, proteomics is mostly applied to fresh frozen tissue samples [15]. However, the storage of frozen tissues is expensive and difficult from a logistical point of view. Formalin fixation followed by impregnation with paraffin results in stable biological specimens, thus making FFPE tissues convenient for handling and long-period storage. Retrospective studies using millions of archived FFPE tissues in hospital represent a valuable information resource of disease epidemiology, pathogenesis and prognosis. Therefore, attention has been increasingly focused on mining proteomic information from FFPE samples.

For years, the efficient protein extraction from FFPE specimens was hindered by the formation of intramolecular and intermolecular cross-links due to formalin fixation. Methylol adducts, Schiff bases, and methylene bridges between the side chains of proteins reduces their solubility and extractability [16]. Recently, a variety of methodological advances for protein extraction have been developed for performing MS on FFPE samples, including improvements in the extraction buffer and extraction environment, which have allowed the identification and quantitation of thousands of proteins from clinical tissue samples. The present report describes our attempts to evaluate and optimize extraction buffers for the robust identification of protein from FFPE rat organ samples.

SDS, as a detergent and denaturant, guarantees enhanced protein recovery [17]; however, interference from trypsin digestion and LC separation has necessitated SDS cleanup steps prior to MS-based proteomic analysis [18]. In the current study, we tested both the detergent removal kit and ethanol precipitation method, both of which led to a certain degree of protein loss. Therefore, we included MS-compatible surfactants, such as Zwittergent and urea, as alternatives to obviate the need for detergent cleanup.

### Advantages and disadvantages of different extraction buffers

All of the five extraction buffers helped identify typical structural and functional proteins after LC-MS/MS analysis. The fluctuation in the extraction efficiency of proteins from different organs indicated which method was superior was possibly tissue specific. For the LMD FFPE specimens, Zwittergent-containing and PEG20000-containing buffers were more proficient than the other buffers, when spectra, peptides, protein number, protein coverage and protein iBAQ were compared. Moreover, Zwittergent was broadly applicable to different tissues. Zwittergent 3–16, as an amphoteric surfactant, retains its activity over a broad pH range, in part due to the presence of basic quaternary ammonium ions and acidic sulfonate ions of equal strength. In the early 1990s, Sekine et al. [19] confirmed that Zwittergent 3–16 was more effective than other detergents, including CHAPS, in retaining the activity of transferase, and maximum activity was obtained at protein concentrations of 0.1%-0.2%. We presume the advantage of Zwittergent might be due to its ability to enhance trypsin digestion. Furthermore, the ionic balance of this detergent prevents protein binding to other anionic or cationic compounds, thereby guaranteeing its extraction, even in small concentrations. In the Mayo Clinic, using Zwittergent-containing buffer, effective protein extraction was achieved from tissue sections as small as 50,000 μm^2^, which was the foundation of subsequent amyloidosis typing [20]. In our center, 200 or more proteins could be identified from microdissected amyloidosis-associated tissue using Zwittergent-containing extraction buffer. Scicchitano et al. [21] extracted 170 proteins from 7 μm thick rat liver sections of 64,000,000 μm^2^, and protein recovery was comparable in our study using even less tissue.

Protein yields from SDS-based extraction buffer were quite satisfactory, although not as good as those from Zwittergent-containing buffer. The addition of PEG20000 improved extraction efficiency, which was consistent with data from a prior study [22]. PEG20000, a high molecular weight carrier substance, reduces the non-specific absorption of protein extracts to surfaces. Since PEG20000 remains in spin ultrafiltration units of a molecular weight cut-off of 10 kDa after trypsin digestion, it does not negatively impact subsequent LC-MS/MS. As indicated in the study by Wisniewski [22], PEG20000 can increase the peptide yield by 30% from samples containing submicrograms to micrograms of protein. For samples containing more than 10 μg proteins, the function of the carrier substance is compromised, which explains why the addition of PEG20000 failed to increase the amount of identified peptides or proteins extracted from FFPE samples in the current study. Taking extraction efficiency and compatibility with MS into consideration, the Zwittergent-containing extraction buffer is the optimal choice for the robust identification of proteins from LMD FFPE rat organ samples.

### Non-LMD versus LMD FFPE samples

In non-LMD FFPE samples, the lowest amounts of proteins were obtained from the Zwittergent-based buffer (mean = 0.6 μg/μL), whereas the protein concentration using the other four buffers reached 1.7–2.4 μg/μL. In current study, protein quantification was determined by the Bradford method. Despite the use of spin ultrafiltration units, different buffer composition might still be partly left in the protein solution, thus interfering the accurate quantification of protein amount. Therefore, protein yields calculated from Bradford method here only provided a rough estimate, compared with iBAQ. As it turned out, the protein iBAQ in the Zwittergent group was not as high as that from buffers 2 and 3. The protein content of the non-LMD specimens was much higher than that of the LMD samples. We assumed that the maximum tissue content that could be resolved by Zwittergent was lower than that using other buffers of the same volume; thus, the protein abundance would increase as the volume of Zwittergent-containing buffer increases. We mixed kidney tissue scraped from one section with 100, 200, 300, 400, and 500 μL Zwittergent buffer. MS results showed that the average protein abundance was highest when the tissue content reached 1.6 mm^3^/mL extraction buffer. For the rat organ FFPE samples in the current study, the tissue content was as high as 6.4 mm^3^/mL buffer, which means the lower protein iBAQ of the Zwittergent group might be explained by inadequate volume of extraction buffer. Buffers 2, 3, and 4 shared a large number of overlapping proteins, which might be explained by the fact that SDS was the common constituent in these three kinds of extraction buffers. Taking spectra, identified unique proteins, overlapping proteins and protein coverage into consideration, Zwittergent was still a good alternative for protein recovery. Regardless of whether the samples were LMD or non-LMD, the extraction efficiency of the urea-containing buffer was most frustrating due to its limited lytic strength.

### Limitations of the current study

Aside from the type of extraction buffer, many pre-analytical and analytical factors impact protein recovery. For example, protein extraction from tissue surrogates is sensitive to the pH of the recovery buffer [23], perhaps related to the pI of the protein within the tissue. Since it was shown that buffers of pH 8.0 or higher give the best extraction yield [24], there is another possibility that alkaline medium improves cross-linking reversal and protein solubility. One limitation of our study is that we did not adjust the pH of the buffers to determine if the buffers functioned at an optimal pH in the different tissues. Moreover, we did not compare the consistency of proteome profiles between fresh frozen and FFPE tissues, which could be another important evaluation criterion of an efficient extraction buffer. Finally, further studies are needed to confirm whether our results are applicable to other rat tissues and human FFPE samples.

## Conclusions

Our objective was to determine the best extraction buffer to use for the accurate identification of the largest number of proteins. For LMD FFPE samples, the Zwittergent-containing buffer was the most effective and efficient with regard to identifying peptides and proteins, whether shared or overlapping. Moreover, unlike SDS, Zwittergent does not impair enzymatic digestion and was well compatible with MS analysis. For non-LMD (slice) samples, Zwittergent 3–16 yielded large numbers of proteins, and the proper volume of buffer might play an important role in increasing protein abundance. As a high molecular weight carrier substance, PEG20000 improves the ability to identify peptides and proteins; however, such an advantage is limited to tissues containing submicrograms to micrograms of protein. Considering its low lytic strength, a urea-containing buffer should not be the first alternative for protein recovery from FFPE specimens. In the future, we believe that the standardization of a method for proteomic analysis of FFPE tissues is absolutely worth pursuing, and extensive applicability to different types of tissues as well as compatibility with enzymatic digestion and MS are two important evaluation criteria.

## Supporting Information

S1 Table The identified top five proteins of five different rat organs from LMD/MS analysis. (DOC)

S2 Table Comparison of protein coverage using five different extraction buffers after LMD/MS analysis. (DOC)

S3 Table The identified top five proteins of slice tissue specimens from LC-MS/MS analysis. (DOC)

S4 Table Comparison of protein coverage from slice tissue specimens using five different extraction buffers after LC-MS/MS analysis. (DOC)

S5 Table The identified proteins and peptide number using different extraction buffers from laser microdissected brain specimens. (XLS)

S6 Table The identified proteins and peptide number using different extraction buffers from laser microdissected heart specimens. (XLS)

S7 Table The identified proteins and peptide number using different extraction buffers from laser microdissected kidney specimens. (XLS)

S8 Table The identified proteins and peptide number using different extraction buffers from laser microdissected liver specimens. (XLS)

S9 Table The identified proteins and peptide number using different extraction buffers from laser microdissected lung specimens. (XLS)

S10 Table The identified proteins and peptide number using different extraction buffers from slice brain specimens. (XLS)

S11 Table The identified proteins and peptide number using different extraction buffers from slice heart specimens. (XLS)

S12 Table The identified proteins and peptide number using different extraction buffers from slice kidney specimens. (XLS)

S13 Table The identified proteins and peptide number using different extraction buffers from slice liver specimens. (XLS)

S14 Table The identified proteins and peptide number using different extraction buffers from slice lung specimens. (XLS)
