# Supplementary material for: Comparison of Different Buffers for Protein Extraction from Formalin-Fixed and Paraffin-Embedded Tissue Specimens
Source: PLoS One. 2015 Nov 18;10(11):e0142650. doi: 10.1371/journal.pone.0142650 (PMC4651363; doi:10.1371/journal.pone.0142650)
Supplement: S1 Table — (DOC) [file pone.0142650.s001.doc]

**S1 Table.** The identified top five proteins of five different rat organs from LMD/MS analysis

| Organ Type | Proteins | Accession Number | Total Spectra | Identified peptides |
| --- | --- | --- | --- | --- |
| Brain | Sodium/potassium-transporting ATPase subunit alpha-3 | P06687 | 38 | 31 |
|  | Tubulin beta-3 chain | Q4QRB4 | 25 | 18 |
|  | Actin, cytoplasmic 1 | P60711 | 23 | 15 |
|  | Tubulin alpha-1A chain | P68370 | 18 | 13 |
|  | Myelin basic protein S | P02688 | 15 | 10 |
|  |  |  |  |  |
| Heart | Myosin-6 | P02563 | 128 | 92 |
|  | Actin, cytoplasmic 1 | P60711 | 36 | 20 |
|  | ATP syntax subunit beta mitochondrial | P10719 | 26 | 16 |
|  | ATP synthase subunit alpha, mitochondrial | P15999 | 19 | 16 |
|  | Tropomyosin alpha-1 chain | P04692 | 16 | 13 |
|  |  |  |  |  |
| Kidney | Actin, cytoplasmic 1 | P60711 | 27 | 17 |
|  | ATP synthase subunit alpha, mitochondrial | P15999 | 18 | 16 |
|  | ATP synthase subunit beta, mitochondrial | P10719 | 18 | 11 |
|  | Sodium/potassium-transporting ATPase subunit alpha-1 | P06685 | 19 | 17 |
|  | Low-density lipoprotein receptor-related protein 2 | P98158 | 15 | 14 |
|  |  |  |  |  |
| Liver | Carbamoyl-phosphate synthase | P07756 | 82 | 54 |
|  | Glutamate dehydrogenase 1, mitochondrial | P10860 | 20 | 16 |
|  | ATP synthase subunit beta, mitochondrial | P10719 | 20 | 13 |
|  | ATP synthase subunit alpha, mitochondrial | P15999 | 19 | 16 |
|  | Actin, cytoplasmic 1 | P60711 | 18 | 13 |
|  |  |  |  |  |
| Lung | Actin, cytoplasmic 2 | P63259 | 29 | 19 |
|  | Vimentin | P31000 | 16 | 14 |
|  | Moesin | O35763 | 12 | 9 |
|  | Tropomyosin | Q63610 | 9 | 9 |
|  | Hemoglobin subunit beta-1 | P02091 | 9 | 7 |
