# Supplementary material for: Comparison of Different Buffers for Protein Extraction from Formalin-Fixed and Paraffin-Embedded Tissue Specimens
Source: PLoS One. 2015 Nov 18;10(11):e0142650. doi: 10.1371/journal.pone.0142650 (PMC4651363; doi:10.1371/journal.pone.0142650)
Supplement: S2 Table — (DOC) [file pone.0142650.s002.doc]

**S2 Table.** Comparison of protein coverage using five different extraction buffers after LMD/MS analysis

|  | Brain |  | Heart |  | Kidney |  | Liver |  | Lung |  |
| --- | --- | --- | --- | --- | --- | --- | --- | --- | --- | --- |
|  | Mean | Median | Mean | Median | Mean | Median | Mean | Median | Mean | Median |
| Buffer 1 | 9.98% | 6.69% | 12.7% | 7.49% | 13.06% | 8.54% | 10.07% | 5.29% | 12.11% | 9.10% |
| Buffer 2 | 4.73% | 2.62% | 8.70% | 4.29% | 7.89% | 5.44% | 9.41% | 6.19% | 7.54% | 4.15% |
| Buffer 3 | 7.11% | 4.23% | 8.41% | 4.01% | 6.44% | 3.73% | 8.51% | 5.56% | 5.73% | 2.42% |
| Buffer 4 | 10.39% | 7.80% | 14.2% | 9.36% | 8.29% | 4.78% | 8.93% | 5.55% | 9.20% | 5.76% |
| Buffer 5 | 3.73% | 2.45% | 5.50% | 3.10% | 3.32% | 1.78% | 3.67% | 2.72% | 3.60% | 0.58% |
