# Supplementary material for: Comparison of Different Buffers for Protein Extraction from Formalin-Fixed and Paraffin-Embedded Tissue Specimens
Source: PLoS One. 2015 Nov 18;10(11):e0142650. doi: 10.1371/journal.pone.0142650 (PMC4651363; doi:10.1371/journal.pone.0142650)
Supplement: S3 Table — (DOC) [file pone.0142650.s003.doc]

**S3 Table.** The identified top five proteins of slice tissue specimens from LC-MS/MS analysis

| Organ Type | Proteins | Accession Number | Total Spectra | Identified peptides |
| --- | --- | --- | --- | --- |
| Brain | Tubulin alpha-4A chain | Q5XIF6 | 103 | 28 |
|  | Tubulin beta-3 chain | Q4QRB4 | 94 | 43 |
|  | Actin, cytoplasmic 2 | P63259 | 83 | 28 |
|  | Sodium/potassium-transporting ATPase subunit alpha-3 | P06687 | 80 | 46 |
|  | Isoform 5 of Myelin basic protein S | P02688 | 71 | 25 |
|  |  |  |  |  |
| Heart | Myosin-6 | P02563 | 209 | 125 |
|  | Actin, aortic smooth muscle | P62738 | 138 | 35 |
|  | ATP synthase subunit beta, mitochondrial | P10719 | 129 | 27 |
|  | ATP synthase subunit alpha, mitochondrial | P15999 | 99 | 33 |
|  | Myosin light chain 3 | P16409 | 79 | 18 |
|  |  |  |  |  |
| Kidney | Cluster of Actin, cytoplasmic 2 | P63259 | 64 | 25 |
|  | ATP synthase subunit beta, mitochondrial | P10719 | 59 | 21 |
|  | ATP synthase subunit alpha, mitochondrial | P15999 | 46 | 22 |
|  | Major urinary protein | P02761 | 46 | 10 |
|  | 60 kDa heat shock protein, mitochondrial | P63039 | 30 | 17 |
|  |  |  |  |  |
| Liver | Carbamoyl-phosphate synthase, mitochondrial | P07756 | 226 | 79 |
|  | ATP synthase subunit beta, mitochondrial | P10719 | 64 | 22 |
|  | Cluster of Betaine--homocysteine S-methyltransferase 1 | O09171 | 57 | 21 |
|  | 60 kDa heat shock protein, mitochondrial | P63039 | 55 | 27 |
|  | Cluster of Actin, cytoplasmic 2 | P63259 | 45 | 19 |
|  |  |  |  |  |
| Lung | Cluster of Actin, cytoplasmic 2 | P63259 | 176 | 37 |
|  | Cluster of Tubulin beta-4B chain | Q6P9T8 | 43 | 21 |
|  | Cluster of Hemoglobin subunit beta-1 | P02091 | 43 | 11 |
|  | Myosin-9 | Q62812 | 39 | 24 |
|  | ATP synthase subunit beta, mitochondrial | P10719 | 35 | 18 |
