# Supplementary material for: Comparison of Different Buffers for Protein Extraction from Formalin-Fixed and Paraffin-Embedded Tissue Specimens
Source: PLoS One. 2015 Nov 18;10(11):e0142650. doi: 10.1371/journal.pone.0142650 (PMC4651363; doi:10.1371/journal.pone.0142650)
Supplement: S4 Table — (DOC) [file pone.0142650.s004.doc]

**S4 Table.** Comparison of protein coverage from slice tissue specimens using five different extraction buffers after LC-MS/MS analysis

|  | Brain |  | Heart |  | Kidney |  | Liver |  | Lung |  |
| --- | --- | --- | --- | --- | --- | --- | --- | --- | --- | --- |
|  | Mean | Median | Mean | Median | Mean | Median | Mean | Median | Mean | Median |
| Buffer 1 | 15.37% | 9.20% | 17.98% | 12.06% | 16.55% | 9.33% | 13.18% | 7.20% | 12.33% | 4.64% |
| Buffer 2 | 11.55% | 6.59% | 14.37% | 6.93% | 12.62% | 7.51% | 14.05% | 8.51% | 10.54% | 6.24% |
| Buffer 3 | 11.40% | 6.52% | 15.37% | 8.41% | 12.30% | 8.04% | 14.85% | 9.85% | 10.71% | 6.47% |
| Buffer 4 | 9.49% | 4.74% | 13.75% | 6.37% | 7.28% | 4.20% | 11.65% | 6.77% | 11.16% | 7.20% |
| Buffer 5 | 4.46% | 2.48% | 5.25% | 3.00% | 3.30% | 1.19% | 1.77% | 0.00% | 3.98% | 1.73% |
